# Short-Term Surgical Outcome for Vestibular Schwannoma in Sweden: A Nation-Wide Registry Study

**DOI:** 10.3389/fneur.2019.00043

**Published:** 2019-01-29

**Authors:** Jiri Bartek Jr., Petter Förander, Erik Thurin, Theresa Wangerid, Roger Henriksson, Göran Hesselager, Asgeir Store Jakola

**Affiliations:** ^1^Department of Neurosurgery, Karolinska University Hospital, Stockholm, Sweden; ^2^Department of Clinical Neuroscience and Department of Medicine, Karolinska Institutet, Stockholm, Sweden; ^3^Department of Neurosurgery, Copenhagen University Hospital Rigshospitalet, Copenhagen, Denmark; ^4^Department of Neurology, Sahlgrenska University Hospital, Gothenburg, Sweden; ^5^Department of Neurology, Capio St. Göran Hospital, Stockholm, Sweden; ^6^Regional Cancer Centre Stockholm/Gotland, Stockholm, Sweden; ^7^Department of Radiation Sciences and Oncology, University of Umeå, Umeå, Sweden; ^8^Department of Neurosurgery, Uppsala University Hospital, Uppsala, Sweden; ^9^Department of Neurosurgery, Sahlgrenska University Hospital, Gothenburg, Sweden; ^10^Institute of Neuroscience and Physiology, University of Gothenburg, Sahlgrenska Academy, Gothenburg, Sweden; ^11^Department of Neurosurgery, St. Olavs University Hospital, Trondheim, Norway

**Keywords:** vestibular schwannoma, neurosurgery, outcome, complications, stereotactic radiosurgery, hematoma, infection, neurological deficit

## Abstract

**Background:** Vestibular Schwannoma (VS) is a benign neoplasm arising from the 8th cranial nerve, with surgery one of the treatment modalities. In a nation-wide registry study, we describe the baseline, treatment characteristics, and short-term outcome in patients surgically treated for VS.

**Methods:** We performed a nationwide study with data from the Swedish Brain Tumor Registry (SBTR) for all adults diagnosed with VS 2009–2015. Patient symptoms, tumor characteristics, and postoperative complications were analyzed.

**Results:** In total 348 patients underwent surgery for VS. Mean age was 50.6 ± 14.5 years and 165 patients (47.4%) were female. The most common symptom was focal neurological deficit (92.0%), with only 25 (7.2%) being asymptomatic prior to surgery, and 217 (63.6%) had no restriction in activity. Following surgery, 100 (28.7%) patients developed new deficit(s). In terms of postoperative complications; 11 (3.2%) had a hematoma, 35 (10.1%) an infection, 10 (2.9%) a venous thromboembolism, and 23 (6.6%) had a reoperation due to complication. There were no deaths within 30-days after surgery. When grouped according to tumor size (< 4 vs. ≥4 cm), those with ≥4 cm tumors were more often males (*p* = 0.02), had more often ICP related symptoms (*p* = 0.03) and shorter time from imaging to surgery (*p* < 0.01). Analysis of the younger (< 65 years) vs. elderly (≥65 years) revealed no difference in outcome except increased 1-year mortality (*p* = 0.002) in elderly.

**Conclusion:** In this nation-wide registry-study, we benchmark the 30-day complication rate after VS surgery as collected by the SBTR. Further, we present the current neurosurgical outcome data from both VS smaller than 40 mm compared to larger tumors, as well as younger vs. elderly VS patients. Since surgical decision making is a careful consideration of short term risk vs. long term benefit, this information can be useful in clinical decision making.

## Introduction

Vestibular Schwannomas (VS) are slow-growing primarily benign tumors of the 8th cranial nerve (1, 2). Patients with VS often present with symptoms related to the 8th cranial nerve such as hearing loss, tinnitus, vertigo or balance difficulties (3). However, VS can also be incidental findings in otherwise asymptomatic patients, a number on the rise due to increased availability of magnetic resonance imaging (4–6).

In VS, surgery is one of the treatment modalities, with the possibility of complete tumor removal with limited risk of recurrence (7). Nevertheless, the surgical procedure is associated with a considerable risk of facial nerve palsy and hearing loss. Intraoperative monitoring and surgical treatment aimed at sparing the cochlear portion of the 8th cranial nerve (so called hearing preservation surgery) is an option, but the results are varied, and clearly dependent on the level of preoperative hearing and tumor size (8–10). Stereotactic radiosurgery (SRS) is an alternative to surgery in VS < 4 cm, which is the often-quoted upper size limit for SRS, especially advocated in patients that might be “unfit” to go through general anesthesia and open surgery (i.e., elderly and those with significant comorbidity) (11–16). Finally, patients with small non-growing tumors might not need treatment at all, with serial imaging and a wait-and-scan approach being the first choice in management (17–19). Thus, decision-making in patients with VS is complex and benchmarking short-term national results of VS surgery is of relevance, since we typically weigh short-term risks against long-term benefit. In addition, a larger national cohort may be used to explore clinically relevant subgroups. Finally, national results may be used to compare against other population-based results to evaluate standard of care.

Thus, the aim of this Swedish nation-wide registry-based study was to explore treatment patterns and short-term outcome within 30-days after surgery for VS, with subgroup analysis according to tumor size and age.

## Materials and Methods

### The Swedish Brain Tumor Registry

The SBTR is a regionally based registry of adult (18 years or older) patients with diagnosed brain tumors. In the Swedish health care system, there are six different regions that provide neurosurgical care to patients with tumors in the central nervous system (CNS), with VS surgery centralized to 5 regions only. All regions report data to SBTR, however the level of coverage has varied between the different regions over time. The variables are assessed and reported to the register by health care personnel. For further details of the registry, see Asklund et al. (20). The highest reported regional number of VS per given year was 17, indicating that none of the regional centers are truly high-volume VS centers.

### Definition of Variables

All regions in Sweden report to the SBTR data concerning baseline characteristics, lead times and outcomes following surgery. The variables registered in the SBTR are described in detail in Table 1.

**Table 1:** Definitions of variables as registered in the Swedish brain tumor registry.

**Variable**	**Definition**
Age	Years at time of diagnosis
Sex	Male or female
Symptoms at diagnosis/presentation	Asymptomatic (yes/no) Focal deficit (yes/no) ICP related (e.g., headache, cognition) (yes/no)
WHO performance status prior to surgery	Grade 0-Fully active, able to carry on all pre-disease performance without restriction Grade I-Restricted in physically strenuous activity but ambulatory and able to carry out work of a light or sedentary nature, e.g., light house work, office work Grade II-Ambulatory and capable of all self-care but unable to carry out any work activities. Up and about more than 50% of waking hours Grade III-Capable of only limited self-care, confined to bed or chair more than 50% of waking hours Grade IV-Completely disabled. Cannot carry on any self-care. Totally confined to bed or chair Grade V-Dead
Date of imaging diagnosis	dd.mm.yyyy
ICD codes used for location only	C72.4 (vestibular schwannoma) C72.5 (cranial nerve NOS)
Laterality	Left/right/bilateral
Largest diameter of tumor prior to surgery	< 4 cm 4–6 cm > 6 cm
Type of surgery	Biopsy or resection
Extent of tumor removal (surgeon evaluated)	Partial, subtotal, or gross-total removal
Date of surgery	dd.mm.yyyy
New or worsened focal deficit within 30 days	Yes/no
Any infection within 30 days	Yes/no
Any VTE within 30 days	Yes/no
Any hematoma within 30 days	Yes/no
Complication leading to reoperation within 30 days	Yes/no
Date of discharge neurosurgical department	dd.mm.yyyy
Histopathology	SNOMED code 9560/0

*ICP, Intracranial Pressure; WHO, World Health Organization; VTE, Venous Thromboembolism; SNOMED, Systematized Nomenclature Of Medicine*.

### Definition of Cohort

Using the SBTR, we aimed to include all patients with VS surgically treated in Sweden from 2009 through 2015 to provide actuality of the current neurosurgical practice. To be included the SNOMED code 9560/0 (Schwannoma) was mandatory. We also combined with topographical codes indicating location at vestibular nerve (C72.4) or cranial nerve NOS (C72.5) to ensure relevant inclusion as done by others (21). In our study we have used data from regions where the total registration for all tumor entities was 80% or more for any given year to provide population-based data. Registration rate was defined as percentage of diagnoses in the SBTR that corresponds to diagnoses reported to the compulsory National Cancer Registry. For this reason, in one region, we used data from 2012 and 2013 only, but for all other regions the inclusion period for this study was from 2009 to 2015. In subgroup analyses we divided the cohort with respect to tumor size, extent of tumor removal, age and gender. For the age variable we used a cut-off of 65 as done previously to separate younger and elderly patients with VS (22–24).

### Statistics

All analyses were done with SPSS, version 24.0 (Chicago, IL, USA). Statistical significance level was set to *p* < 0.05. All tests are two-sided. Central tendencies are presented as means ± SD, or median and interquartile range if skewed. Categorical data was analyzed with Pearson's chi-square test.

### Ethics Statement

This project was approved by the regional ethical committee in Western Sweden and by the registry holder, Dnr 363-17. The ethics committee has waived the need for written informed consent due to the nature (registry-based) of the study.

## Results

### Baseline Characteristics

In total, 348 patients underwent surgery being assigned the SNOMED code 9560/0 confirming a diagnosis of schwannoma. According to the ICD-10 code of location, 257 (73.9%) were C72.4 confirming vestibular schwannoma and 91 (26.1%) was C72.5 only indicating schwannoma location in relation to a cranial nerve, but where also the majority of these are expected to be vestibular schwannoma.

Mean age was 50.6 ± 14.5 years and 165 patients (47.4%) were female. Most patients presented with a focal deficit (92.0%), with only 25 (7.2%) asymptomatic prior to surgery. Nevertheless, the majority [217 (63.6%)] had no restriction in activity (WHO 0 performance status). Most tumors [252 (85.7%)] were classified as < 4 cm, with 37 (12.6%) sized 4–6 cm and 5 (1.7%) > 6 cm. There was no significant difference between laterality (*p* = 0.19).

The surgery was gross total in most patients [231 (66.4%)], with 100 (28.7%) patients developing a new postoperative deficit. In terms of postoperative complications; 11 (3.2%) had a hematoma, 35 (10.1%) an infection, 10 (2.9%) a venous thromboembolism and 23 (6.6%) had a reoperation due to complication. There were no deaths within 30-days after surgery, with four deaths (1.1%) registered after 1 year. For further details see Tables 1, 2. We created a multivariable model of preoperative factors to predict the most common adverse events related to surgery in our cohort (i.e., new deficit, infection and reoperation due to complication). The model consisted of the possible predictors age, sex, tumor size, and functional level. Based on these parameters, the only independent predictor was that higher age was associated with less infection (*p* = 0.049, HR 0.97, 95% CI 0.93–1.0).

**Table 2:** Baseline characteristics, including comparison of patients with VS according to tumor size.

	**Total (*N* = 348)**	< 4 cm (*N* = 252)^*^	≥ 4 cm (*N* = 42)^*^	*p*-Value^**^
Age, mean (SD)	50.6 (14.5)	50.7 (14.3)	48.5 (16.8)	0.38
Female, *n* (%)	165 (47.4)	127 (50.4)	13 (31.0)	0.02
Preop MRI, *n* (%) Missing, *n* = 1	343 (98.8)	248/251 (98.8)	42 (100)	0.48
Tumor size, *n* (%)				–
< 4 cm	252 (85.7)	(100)	0	
4–6 cm	37 (12.6)	0	37 (88.1)	
>6 cm	5 (1.7)	0	5 (11.9)	
Missing, *n* = 54				
Laterality				0.19
Right, *n* (%)	155 (47.3)	111/243 (45.7)	19/36 (52.8)	
Left, *n* (%)	171 (52.1)	131/243 (53.9)	16/36 (44.4)	
Bilateral, *n* (%)	2 (0.6)	1/243 (0.4)	1/36 (2.8)	
Missing, *n* = 20				
Asymptomatic, *n* (%)	25 (7.2)	19 (7.5)	2 (4.8)	0.52
Focal deficit, *n* (%) Missing, *n* = 21	201 (92.0)	224/237 (94.5)	38/40 (95.0)	0.90
ICP related, *n* (%)	91 (27.8)	46/237 (19.4)	14/40 (35.0)	0.03
Missing, *n* = 21				
Performance status, *n* (%)				0.06
0	217 (63.6)	163/249 (65.5)	24 (57.1)	
1	73 (21.4)	53/249 (21.3)	10 (23.8)	
2	44 (12.9)	30/249 (12.0)	5 (11.9)	
3	6 (1.8)	3/249 (1.2)	2 (4.8)	
4	1 (0.3)	0 –	1 (2.4)	
Missing, *n* = 7				
Imaging diagnosis to surgery, median, months (IQR)	26 (13–68)	26 (16–68)	12 (6–36)	< 0.01

*MRI, magnetic resonance imaging; ICP, intracranial pressure*.

* *Missing reported in the group as a whole, fraction reported in analyses of size when missing data for the specific variable*.

** *p-Value comparison between small and larger vestibular schwannomas*.

### Comparison Between GTR vs. Partial and Subtotal Resections

We analyzed baseline and outcome in patients undergoing GTR compared to partial and subtotal resections. The only significant difference in baseline was that GTR was less common in patients with ICP related symptoms where 50.5% underwent GTR compared to 72.9% in those without ICP related symptoms (*p* < 0.001). This finding was not obviously associated with tumor size (*p* = 0.85). In outcome the only difference was that fewer patients with GTR experienced postoperative hematoma (1.7% compared to 6.0%, *p* = 0.03).

### Comparison According to Tumor Size

When grouped according to tumor size (< 4 cm vs. ≥4 cm) there was a significant difference among groups in gender (*p* = 0.02), ICP related symptoms (*p* = 0.03), and time from diagnosis to surgery (*p* < 0.01). No significant difference was noted with respect to the intraoperative and postoperative variables. For further details see Tables 2, 3.

**Table 3:** Intraoperative and postoperative variables, including comparison according to tumor size.

	**Total (*N* = 348)**	< 4 cm (*N* = 252)^*^	≥ 4 cm (*N* = 42)^*^	*p*-Value^**^
Tumor removal, *n* (%)				0.84
Partial	97 (27.9)	76 (30.2)	13 (31.0)	
Subtotal	20 (5.7)	2 (0.8)	0	
Gross total	231 (66.4)	174 (69.0)	29 (69.0)	
New deficit, *n* (%)	100 (28.7)	66 (26.2)	13 (31.0)	0.52
Hematoma, *n* (%)	11 (3.2)	2 (0.8)	1 (2.4)	0.34
Reoperation due to complication, *n* (%)	23 (6.6)	15 (6.0)	3 (7.1)	0.77
Infection, *n* (%)	35 (10.1)	20 (7.9)	4 (9.5)	0.73
VTE, *n* (%)	10 (2.9)	7 (2.8)	0	0.27
30 day mortality, *n* (%)	0	–	–	
1-year mortality, *n* (%)	4 (1.1)	2 (0.8)	1 (2.4)	0.34

*VTE, venous thromboembolism*.

* *54 missing with respect to size*.

** *p-Value comparison between small and larger vestibular schwannomas*.

### Comparison Between Younger and Elderly VS Patients

When grouped according to patient age (younger, < 65 years vs. elderly, ≥65 years) a significant difference in baseline characteristics was found, with worse functional status, exemplified with performance status grade 0 in 66.5% of younger and 49.1% of elderly patients (*p* = 0.03). This was not accompanied by any significant differences in baseline characteristics such as tumor size or symptoms. There was no difference in outcome between groups, except in 1-year mortality (*p* = 0.002). Further subgroup analyses of patients with VS size < 4 cm didn't reveal any differences in outcome when younger vs. elderly were compared. For further details see Tables 4, 5.

**Table 4:** Comparison of outcome in younger (< 65 years) vs. elderly (65 years or older) VS patients.

	**18–64 years (*N* = 289)**	**65 years or older (*N* = 58)**	***p*-Value**
Size, *n* (%)			0.57
< 4 cm	207 (85.5)	44 (86.5)	
4–6 cm	30 (12.4)	7 (13.5)	
>6 cm	5 (2.1)	0	
Missing = 54			
New deficit, *n* (%)	84 (29.1)	16 (27.1)	0.76
Infection, *n* (%)	33 (11.4)	2 (3.4)	0.06
Hematoma, *n* (%)	8 (2.8)	3 (5.1)	0.35
Reop complication, *n* (%)	21 (7.3)	2 (3.4)	0.28
VTE, *n* (%)	10 (3.5)	0	0.15
1-year mortality, *n* (%)	1 (0.3)	3 (5.1)	0.002

*VTE, venous thromboembolism*.

**Table 5:** Comparison of outcome in younger (< 65 years) vs. elderly (65 years or older) patients with VS size < 4 cm.

	**18–64 years (*N* = 207)**	**65 years or older (*N* = 44)**	***p*-Value**
New deficit, *n* (%)	53 (25.6)	16 (28.9)	0.65
Infection, *n* (%)	18 (8.7)	2 (4.4)	0.34
Hematoma, *n* (%)	2 (1.0)	0	0.51
Reop complication, *n* (%)	14 (6.8)	1 (2.2)	0.24
VTE, *n* (%)	7 (3.4)	0	0.21
1-year mortality, *n* (%)	1 (0.5)	1 (2.2)	0.23

*VTE, venous thromboembolism*.

### Comparison Between Gender

We further analyzed baseline characteristics and outcome separately according to gender. The only difference was a difference in laterality of tumors, with majority of female patients with tumor on the left side (57.7%) compared males with majority on the right side (52.9%, *p* = 0.04). There were no differences between genders in term of outcome.

## Discussion

In this nationwide registry-based study spanning from 2009 to 2015, we describe the baseline and treatment characteristics as well as the 30-day complication rate after VS surgery as collected by the SBTR. Further, we present the current surgical outcome data from VS patients according to tumor size, as well as other clinical relevant subgroups. The results illustrate what is to be expected from current neurosurgical practice in terms of tumor removal and short-term surgical complications and can be helpful in guiding physicians and patients alike. The risk willingness may differ significantly between patients, and although long-term results are of outmost importance, the path toward the result can be different across the different therapeutic approaches. Since decision-making in patients with VS is complex, we think our results contribute significantly to elucidating on the “surgical pathway.”

Register-based results on outcome after VS surgery have been published previously, albeit most reports are older and may not be representative for the current treatment practice (25). Another limitation for the generalizability of previous results is that some are based on non-consecutive and non-population-based material (i.e., patients only treated in selected academic centers) (26–29). Finally, the SBTR is a generic brain tumor registry and we report on the surgical outcome in more general terms, while some of the earlier registry studies have primarily focused on functional outcome such as hearing preservation and/or facial nerve function (18).

The baseline characteristics in our material show a mean age of 50.6 years at diagnosis and this is comparable to the most recent surgical register-based publication (26), while lower than the average of 58 years reported from a register-based study in Denmark (18) although the Danish study also included patients with wait-and-scan regimen and this may explain the difference in age. Given the estimated incidence of a little more than 6/100.000 per year in Sweden, where the population increased from just above 9 million to approximately 9.8 million during the seven-year study period (Statistics Sweden: www.scb.se), surgery appears to be the main treatment modality (21). Still, there is naturally a selection of patients with VS treated with surgery, unfortunately we are unable to elaborate on this selection process since the registry only includes histopathological verified cases.

It has been observed that male VS patients have larger tumors and a higher incidence of hearing loss at presentation compared to female VS patients (30). It has also been noted there is a larger number of females than males receiving surgical treatment (26). In our material 50.4% of patients with small tumors were female, but for patients with larger tumors (≥ 4 cm) only 31%, (*p* = 0.02) were female. It is unclear if this difference depends on tumor biology, females seeking medical care earlier, choosing surgery over other treatment modalities or wait-and-scan or other systematic differences in treatment. In our material we could not find any apparent time trend association of differences between gender (data not shown).

Interestingly, in 63.6% of the patients the VS had no restriction on the daily activity, although 92% had focal deficit(s), which can be interpreted as the focal deficits being sufficiently mild in order not to restrict daily activity. This fits well with most symptoms often related to some degree of unilateral hearing loss and tinnitus thus not influencing the physical activity level significantly, while severe vertigo or balance difficulties are unusual, but when present affect quality of life to a higher degree (3).

Gross tumor removal was achieved in 69% of patients. In the first three years (2009–2011) GTR was reported in 71.2% while in the last 3 years (2013–2015) GTR was reported in 57.0% (data not shown), perhaps an indication of increased focus on preservation of cranial nerve function where SRS may be used to treat small remnants following subtotal resection (31–37). Nevertheless, there was no apparent difference in postoperative deficit in the time periods above (23.7% in early period vs. 27.6% in late period). There was no difference in the degree of tumor removal according to tumor size, neither was there any difference in the frequency of new deficits among patients in the two groups (*p* = 0.52). The per-operative mortality in our material was 0%, like previous reports reporting on a very low perioperative mortality of < 0.5% (8), while still others report of up to 1% mortality in the most difficult cases (38).

In terms of complication frequency, it is difficult to compare our results to those of others, since the definition of an “complication” is not uniform, nor is the registration once the patient has been discharged. For instance, while the infection frequency of 10% reported by the SBTR is high compared to the 0.2% reported by Hatch et al. (26), the 0.2% represent surgical site infections only, not including urinary tract infection, pneumonia, sepsis etc., demonstrating the need for standardized variables across studies. Similarly, in SBTR postoperative hematoma was registered in 3.2% of patients, which is considerably higher than the 1.2% recently reported by others (26). The definition and screening for postoperative hematoma is not uniform and it is unclear if the reported number represents a true difference in complication frequency, but this could be a topic for improving quality in Sweden. An association with subtotal removal was seen, and this may indicate a tendency of tumor remnant to cause bleeding or quality of surgery since both postoperative hematoma and tumor removal may be associated with experience and/or surgical technique. Venous thromboembolism (VTE) frequency of 2.9% ought to be comparable, since the VTE definition is almost universal. As such, our VTE frequency is higher than the 0.8% reported recently (26)—the reason for this can only be speculated upon, but could at least be associated to the conservative long-lasting withdrawal of antiplatelet and anticoagulation in neurosurgical patients in Sweden, which has recently been demonstrated in chronic subdural patients (39). Differences in mobilization regimes and screening for VTE may also play a role. Unfortunately, we have no data on duration of surgery, but this could be an issue since it is a strong association between longer lasting surgeries and postoperative complications (40, 41). Also, the SBTR variables reoperation due to complication encompasses both CSF-leak repairs as well as evacuation of a post-operative hematoma, and as such a frequency of 6.6% is comparable with the remaining literature (26). Finally, our multivariable model did not reveal any association between preoperative factors and complications (i.e., new deficit, infection and reoperation due to complication) besides the probable spurious association between higher age and less infections.

There are two reasons for dividing VS according to size when comparing results, one being previously reported higher complication frequency in surgically treated large VS (38, 42–44), and the second being the possibility of SRS for those VS in need of treatment (not suitable for the wait-and-scan strategy), with an upper limit of the possibility for SRS treatment often quoted as 3, but to a maximum of 4 cm in diameter (13, 16, 45, 46). In terms of tumor control in SRS treated VS, it is often reported to be >90%, and while some report on lower tumor control in large VS, others did not find any correlation between tumor control and VS size (47). The complication frequency of SRS of VS is mainly related to dose and volume (48–50) and while tumor control is reported to be similar in microsurgery, the risk of focal complication is often reported to be higher in microsurgery (28, 51, 52).

An important aspect to discuss in this context is the rising number of elderly patients, especially since open surgery and tumor removal has been reported to be associated with worse outcome in comparison to younger patients (22, 53). In our material, we didn't find any difference in outcome between the young and elderly, except increased 1-year mortality among the elderly which may be expected simply due to shorter life expectancy in the elderly.

Even a subgroup analysis on patients potentially amendable for SRS (VS < 4 cm) divided into younger vs. elderly did not show any difference in outcome. In our material, 44 of the 58 elderly patients had VS < 4 cm that were theoretically candidates for SRS, although we don't have individual data to determine actual image size or if potential complicating factors made SRS contraindicated. In these patients, there were almost 30% new deficits after surgery, and although complications did not differ from younger patients in our material, the indication for SRS is perhaps strengthened with expected reduced need of later rescue therapy, and less risk from secondary cancer—a theoretical drawback of SRS, simply by fewer expected life years after treatment. We have no data for VS patients that were treated with SRS, as SBTR only includes histopathological verified VS patients. However, it is possible to speculate that among elderly with smaller VS there is an under-utilization of SRS. The numbers provided in this study are particularly of interest to this cohort when comparing treatment options (Table 5), since prospective studies (level 2 evidence) in literature suggest the superiority of SRS over microneurosurgery in patients with VS < 3 cm in diameter (54, 55).

In summary, a nation-wide report on outcome and 30-day complication rate following VS surgery provides numbers that can be used by physicians and patients when choosing between treatment modalities, and if non-surgical cases (i.e., SRS treated patients) are included in the future, it may also be used for a population-based technique comparison.

### Strengths and Limitations

The SBTR registry is limited to reporting surgically treated VS only, with no patients treated with SRS, or those followed with the wait-and-scan policy have been registered. Limitations of this study further include those typical of registry-based studies with limited details and without possibility to complete missing data since data is provided without identification from the registry holder. Also, there is a lack of variables including audiometric parameters, and i.e., new neurological deficits are not provided in detail, while other variables are standardized into certain categories i.e., tumor diameter registered being divided into < 4 cm, 4–6 cm and >6 cm. The lack of long-term follow-up, for instance if the neurological deficit is transient or permanent, is another limitation. Finally, some variables may be subject to considerable ambiguity (i.e., postoperative hematoma) while others are more robust (i.e., deep vein thrombosis). Strengths include nation-wide population-based inclusion of many patients from a recent period where data is reported in a prospective, continuous and standardized fashion.

## Conclusion

In this Swedish nationwide population-based registry study, we benchmark the 30-day overall complication rate as 16.2% with 28.7% developing new deficits and 6.6% in need of reoperation after VS surgery. Results were similar in patients with VS < 4 cm compared to those > 4 cm, and patients 65 years or older had similar perioperative outcomes compared to younger patients, but a higher 1-year mortality.

## Author Contributions

JB, PF, and AJ: study design, statistical analysis. JB and PF: draft of manuscript. AJ: study supervision. All authors: data interpretation, revision, and approval of manuscript.

### Conflict of Interest Statement

The authors declare that the research was conducted in the absence of any commercial or financial relationships that could be construed as a potential conflict of interest.
